# Recent Progress of Propolis for Its Biological and Chemical Compositions and Its Botanical Origin

**DOI:** 10.1155/2013/697390

**Published:** 2013-04-30

**Authors:** Viviane Cristina Toreti, Helia Harumi Sato, Glaucia Maria Pastore, Yong Kun Park

**Affiliations:** Department of Food Science, College of Food Engineering, State University of Campinas, Monteiro Lobato Street n.80, P.O. Box 6177, 13083-862 Campinas, SP, Brazil

## Abstract

Propolis is the generic name given to the product obtained from resinous substances, which is gummy and balsamic and which is collected by bees from flowers, buds, and exudates of plants. It is a popular folk medicine possessing a broad spectrum of biological activities. These biological properties are related to its chemical composition and more specifically to the phenolic compounds that vary in their structure and concentration depending on the region of production, availability of sources to collect plant resins, genetic variability of the queen bee, the technique used for production, and the season in which propolis is produced. Many scientific articles are published every year in different international journal, and several groups of researchers have focused their attention on the chemical compounds and biological activity of propolis. This paper presents a review on the publications on propolis and patents of applications and biological constituents of propolis.

## 1. Introduction

Propolis is a resinous substance collected by *Apis mellifera *from various tree buds which they then use to coat hive parts and to seal cracks and crevices in the hive [1]. Propolis has been used as a folk medicine since 300 BC [2]. Recently, numerous biological properties of propolis have been reported including cytotoxic, antiherpes, free radical scavenging, antimicrobial, and anti-HIV activities [3–9]. Because of the wide range of biological activities, propolis has recently been extensively used in food and beverages to improve health and prevent diseases [10–12].

The medical application of propolis preparation has led to an increased interest in its chemical compositions and its botanical origins, because so far mainly polyphenol compounds have been identified in propolis collected by *Apis mellifera*. The flavonoids, the main polyphenols in propolis, have been found to be quantitatively or qualitatively variable, depending on the environmental plant ecology [13–16].

## 2. History of Propolis and the Research on Propolis

The word “propolis” is derived from the Greek pro (for “in front of” or “at the entrance to”) and polis (“community” or “city”) and means a substance in defense of the hive. Propolis, or bee glue, is a brownish resinous material collected by worker bees from the leaf buds of numerous tree species like birch, poplar, pine, alder, willow, palm, *Baccharis dracunculifolia*, and *Dalbergia ecastaphyllum* [15, 17, 18]. In order to manufacture propolis, bees may also use material actively secreted by plants or exuded from wounds in plants [18].

**Figure 1:**
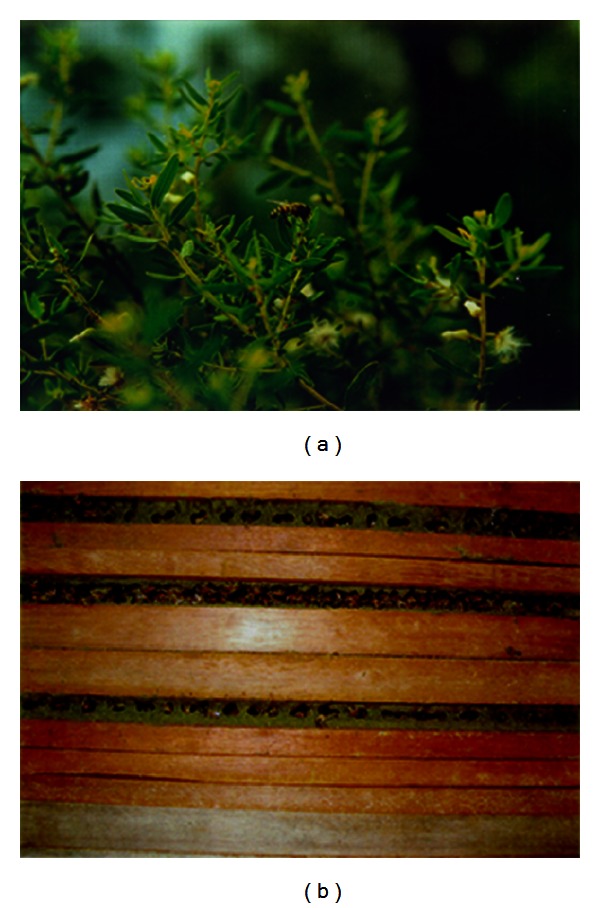
*Apis mellifera* collecting propolis of *Baccharis dracunculifolia* to production propolis in Brazil. (a) *Apis mellifera* collecting leaf apices *Baccharis dracunculifolia*; (b) deposition of green propolis cracks in the hive.

Propolis has been used by man since early times for various purposes as an antiseptic, antioxidant, antiinflammatory, and an adhesive and to seal cracks; to protect wooden and other surfaces. The bees use propolis to repair combs, to strengthen the thin borders of the comb, and to make the entrance of the hive weathertight or easier to defend. Propolis is also used as an “embalming” substance to cover the carcass of a hive invader which the bees have killed but cannot transport out of the hive. The bees cover the invader with propolis and wax, and the remains are left at the bottom or on one of the walls of the hive [2, 19].  Figure 1 illustrates the *Apis mellifera *collecting resinous material from leaf buds of *Baccharis dracunculifolia *(a) and the deposition of green propolis in the hive (b).

In early records are mentioned substances that cast doubt on the use or not of propolis. In Genesis (c. 1700 BC), tzori was taken to Egypt, once with honey and its healing properties are noted three times in Jeremiah. Twice, tzori came from Gilead, but it was not balm of Gilead which is produced from a tree, *Commiphora opobalsamum*. Assis believed that “black wax” referred to in the Egyptian Ebers papyrus (c. 1550 BC) may have been propolis. He also considered that Hebrew tzori was an early word for propolis. This occured six times in the Hebrew scriptures and was usually translated as balm or balsam [19].

Egyptians knew very well the antiputrefactive properties of propolis and used it to embalm cadavers. Greek and Roman physicians Aristoteles, Dioscorides, Pliny, and Galen were familiar with the medicinal properties of propolis. It is not known what methods were used for harvesting it in the ancient world, although writers in Greece and Rome were familiar with it. The Greek *Historia Animalium* referred to a substance *mitys*, which was probably propolis, as “a cure for bruises and suppurating sores.” According to Varro in Rome, propolis was used by physicians in making poultices, and for this reason it brings even a higher price than honey on the Via Sacra [19].

The propolis was employed as an antiseptic and cicatrizant in wound treatment and as a mouth disinfectant, with these uses being perpetuated in the Middle Ages and among Arab physicians. Propolis was also recognized by other peoples unrelated to the Old World civilizations: Incas employed propolis as an antipyretic agent, and the London pharmacopoeias of the seventeenth century listed propolis as an official drug. Between the seventeenth and twentieth centuries, the propolis became very popular in Europe on account of its antibacterial activity [18].

### 2.1. Publications and Patents

The first work indexed by *Chemical Abstracts* on propolis was in 1903, and the first patent was described in 1904 (USA—Composition for treating pins and piano strings). One hundred and nine years after the first publication in the *Chemical Abstract*, the number of publications on propolis reached 3,880 in journal and 2,884 in patents.

**Figure 2:**
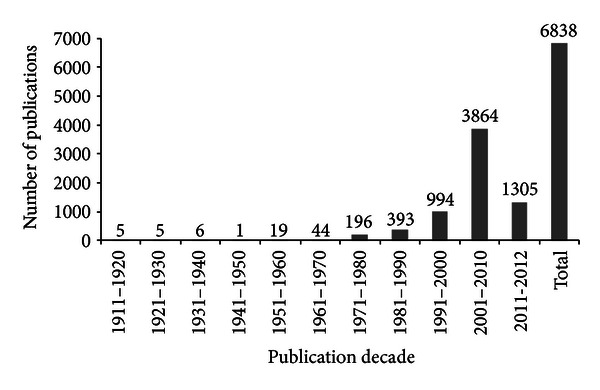
Scientific productivity on propolis between the decades (*Chemical Abstracts*).

Figure 2 shows the number of publications on propolis over the decades; data were obtained by searching the *Chemical Abstracts*. The global interest in propolis research presents two justifications according to Pereira et al. [20], the first to present diverse biological properties and the second by high added value, the sale price of a bottle in Brazil is about 5 to 10 real.

**Figure 3:**
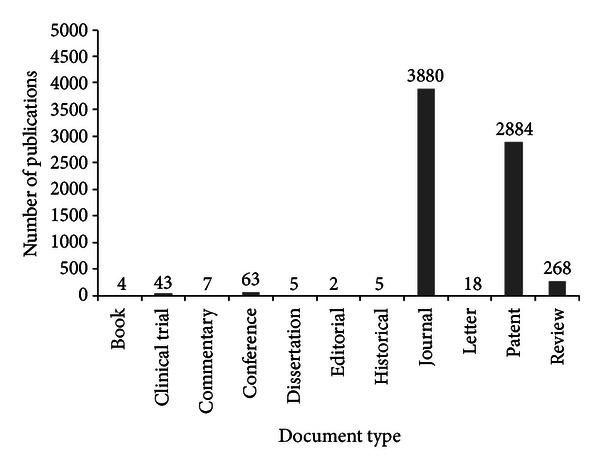
Scientific production on propolis by document type (*Chemical Abstracts*).

The scientific production about propolis by document type is the majority about journal and patents as shown in Figure 3.

The processed patent documents contain highly valuable legal, economic, and technical information; hence, the results obtained from their processing make it possible to obtain highly valuable information to reach conclusions useful as key elements for the design of R&D, technological surveillance, market studies, and marketing strategies [21].

**Figure 4:**
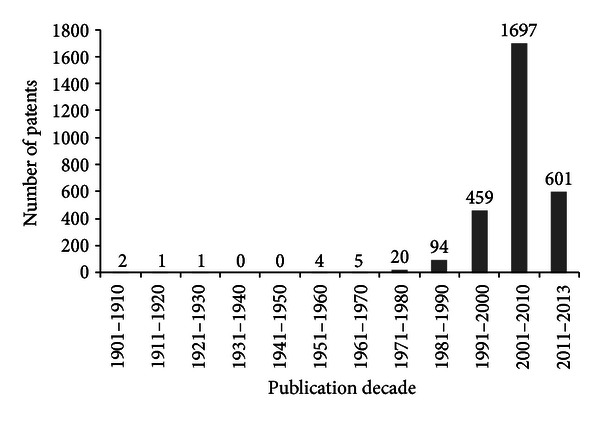
Scientific production on propolis by patents (*Chemical Abstracts*).

Since the first patent was filed and until today, it is possible to see that there was a considerable increase in the number of patents in the last thirty years (Figure 4).

**Figure 5:**
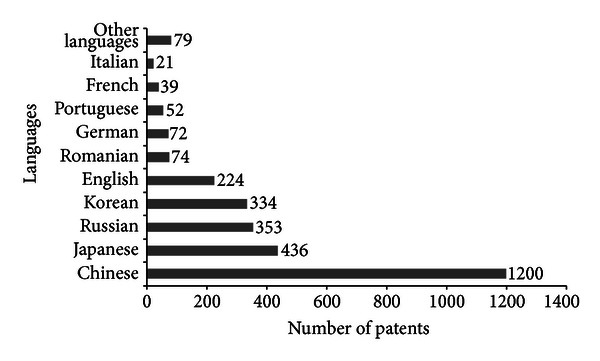
Scientific production on propolis by patents and languages (*Chemical Abstracts*).

According to profile of patents, shown in Figure 5, China, Japan, and Russia are those that hold most of the patents. This fact can be justified by China and Russia being the largest producers of propolis. Today 42% of patents are Chinese (Figure 5), and the first Chinese patent appeared in 1993 (on “Process for production mouth freshener”). The Japanese have 15% of patents, and the first appeared in 1988 (about “Deodorants controlling mouth odor”). The first patent was obtained in 1968 on Russian “Toothpaste” and represented 12% of patents. Brazil deposited its first patent in 1997 on “Dental gel.” Some patents are presented in Table 1.

Suárez et al. [21] noted the trend of patent applications according to application area for the period 1972–2000 and observed a high incidence of products with medicinal and nutraceutical properties and with dermatological applications.

**Figure 6:**
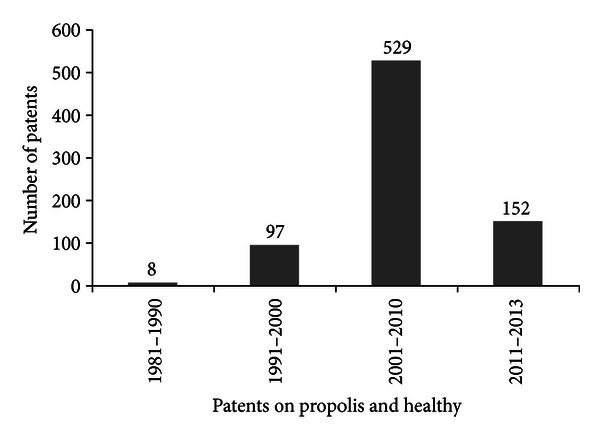
Scientific production on propolis and healthy by patents between the decades (*Chemical Abstracts*).

The scientific production on propolis and healthy patents between the decades in shown in Figure 6.

**Figure 7:**
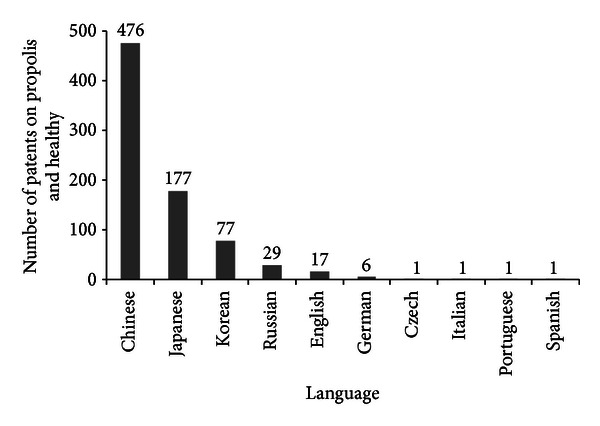
Scientific production on propolis and healthy by patents and languages (*Chemical Abstracts*).

The inventions processed according to their priority country are which shown in Figure 7 in which the highest number of filings corresponds to China (476), followed by Japan (177), Korea and Russian with 77, and 29 and filings, respectively. Japan imports almost all the propolis used in the country: 80% comes from Brazil and 10% from China and other countries, and this is shown in their inventions, since in the analysis carried out to the content of the Japanese inventions we can see that the Brazilian propolis is the target of invention [21].

**Figure 8:**
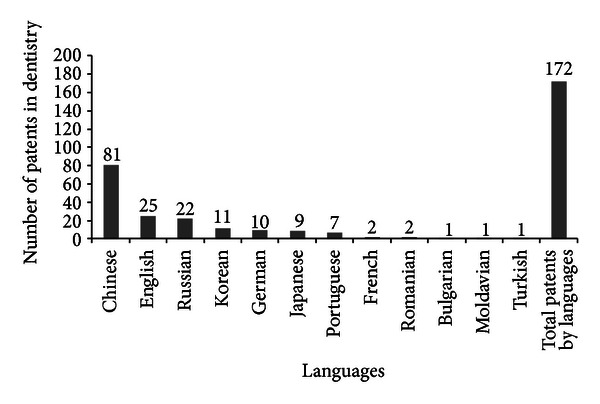
Scientific production on propolis for dental treatment by patents and languages (*Chemical Abstracts*).

Six percent of patents filed by 2012, refers to the use of propolis for dental treatment (Figure 8). According to Pereira et al. [20], this is one of the most widely studied applications of propolis worldwide.

According to the Ministry of Development Industry and Foreign Trade of Brazil [22], the Brazilian export market of propolis in the year of 2012 was 41,721 Kg corresponding about $5,401,643. These values can be observed in Table 2.

Analyzing the data, it is possible to observe that there was a drop in sales in 2011 compared to 2010. Regarding the value of the product, there was an increase of over 50% in 2012 compared to 2010. According to Aga et al. [23], the country that buys Brazilian propolis is Japan, and its extensive use in foods and beverages intended to maintain or improve human health.

## 3. Chemical Composition and Propolis Activity

### 3.1. Chemical Composition

Propolis is a complex resinous mixture which contains approximately 50% of resin and balsam, 30% of wax, 10% of essential and aromatic oils, 5% of pollen, and 5% of impurities [24]. The chemical composition of propolis is highly variable mainly due to the variability of plant species growing around the hive, from which the bees collect the exudates [18, 25–28]. Additionally, propolis composition can vary depending on the seasonality, illumination, altitude, collector type, and food availability and activity developed during propolis exploitation [29–33].

**Figure 9:**
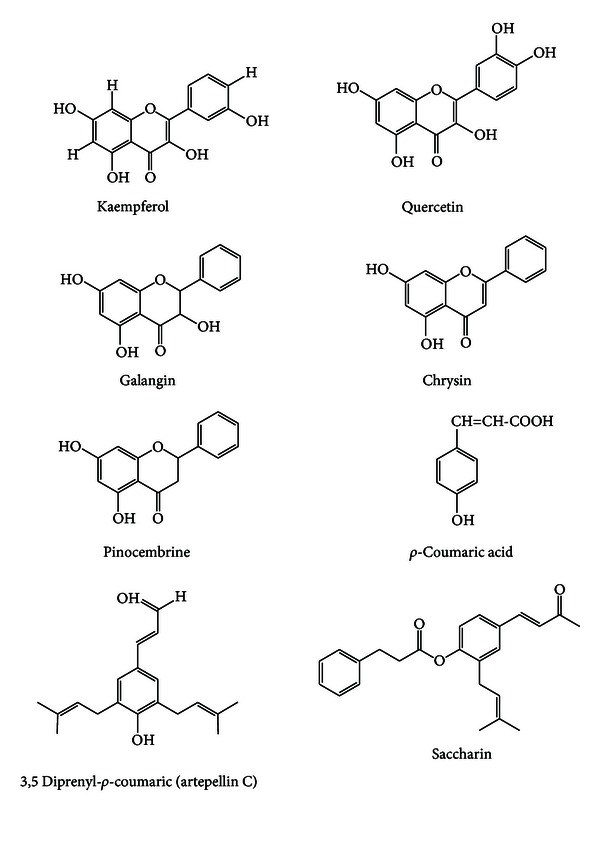
Some typical flavonoids and phenolics present in green propolis.

Much work has been conducted on the chemical composition and biological activities. Until now, more than 300 chemical constituents have been identified in propolis from different regions [34]. The main chemical classes present in propolis are flavonoids, phenolics, and aromatic compounds (Figure 9) [35, 36]. Propolis also contains some volatile oils, terpenes, and bee wax, but these compounds are not believed to contribute as significantly to the chemical properties and effects of propolis [36].

Many analytical methods have been used for separation and identification of propolis constituents. Differents compounds have been identified in ethanol extracts of propolis (Table 3).

### 3.2. Method of Extraction

The method of extraction and solvent can change the chemical composition of propolis extract. Commercial products such as tablets, capsules, ampoules, and syrups are prepared with ethanolic extract of propolis. Methanol is only used for research purposes. Some varieties of propolis have solubility in water, thereby extracting water should also be considered for study [35].

### 3.3. Origin Botanical

The materials available to bees for production of propolis are substances actively secreted by plants as well as substances exuded from wounds in plants: lipophilic materials on leaves and leaf buds, resins, mucilages, gums, lattices, and so forth [37].

The composition of the plant source determines the chemical composition of bee glue, and it is dependent on its geographical location; as a result, its biological activity is closely related to the vegetation native to the site of collection [14, 38, 39].

Bankova [38] discusses the diversity of the chemical composition of propolis and the problem of standardization. The issue is based on the chemical composition of propolis which varies with the plant source collection. Dealing with reliable criteria for chemical standardization of different propolis types is needed, but such generally accepted criteria do not yet exist. There is still a lot of work to be done to achieve standardization of other propolis types. Working with standardization material will allow scientists to connect a particular chemical propolis type to a specific type of biological activity and formulate recommendations.  Table 4 illustrates propolis of different geographic regions and their principal plant sources of chemical compounds.

### 3.4. Classification of Brazilian Propolis

It was found that propolis from several regions of Brazil show different chemical composition, depending on the local flora at the site of collection [40]. The propolis from Brazil was classified in types according to its geographical origin, chemical composition, and source plant as shown in Table 5 [17, 41, 42]. More studies should be done in order to standardize propolis.

### 3.5. Activity of Propolis

It is important to note that most of the latest investigations on new propolis constituents are connected to their biological activity. This information is summarized in Table 6. Some compounds from propolis have antibacterial activity, antitumor activity, and antiinflammatory activity, antioxidative and hepatoprotective action.

According to Bankova et al. [34], relating the chemical constituents of propolis with biological activity enables the standardization of the application of propolis. Kumazawa et. al. [28] report that differences in the chemical composition of propolis from different sources change the spectrum of biological activity of propolis.

Some studies have been conducted correlating chemical composition and biological activity, but no tested compounds were isolated [43–47].

## 4. Application in Medicine and Dentistry

Propolis has been found to have a wide spectrum of biological and pharmaceutical properties and has been demonstrated to have direct antimicrobial effects *in vitro* [48]. Some recent studies suggested that propolis can be used in medicine and dentistry. Tables 7 and 8 illustrate some studies that show the application of propolis in medicine and dentistry.

## 5. Conclusions

Propolis has been used extensively as a folk medicine because of its special chemical components, strong pharmacological, properties and low toxicity. This wide spectrum of therapeutic effects makes propolis a potential candidate in several clinical scenarios. Clinical studies are now also in progress to verify the effects of propolis in the prevention and treatment of diseases.

The application of propolis is mostly in the drug or food manufacture in the form of mixtures. Current opinion is that the use of standardized preparations of propolis is safe and less toxic than many synthetic medicines, but the components of propolis are variable, and it is difficult to standardize and apply propolis. Robust manufacturing processes, standardized quality controls, and good design clinical trials are all critical steps in verifying these claims.

## Figures and Tables

**Table 1:** Relationship of some of the 2,884 international patents (*Chemical Abstracts*).

Year	Country	Title	Register
1904	USA	“Composition for treating piano pins and strings”	US767499
1920	Unavailable	“Paint vehicles”	GB146986
1921	USA	“Waterproof paint”	US1401261
1952	Unavailable	“Tooth paste and oral disinfectant”	AT172063
1969	USSR	“A dental elixir”	SU240182
1979	Romania	“A powder containing soluble propolis”	RO67036
1990	Japan	“Processed food containing propolis”	JP02154652
1995	USA	“Treatment of acne/Pharmaceutical compositions for treatment of acne containing extracts of propolis, verbascum, etc”	US5399349
2009	Turkey	“Use of propolis as base material under dental inlays and as filling material for root canals”	TR2009000486
2011	Turkey	“Extract of propolis and Cramp Bark (*Viburnum opulus*) with high content of phenolic compounds useful as natural remedy”	TR2011000075
2012	Korea	“Method for manufacturing functional food containing propolis”	KR2012136769

**Table 2:** Brazilian propolis export market [49].

Year	Quantity export (Kg)	Value (U$S)	Value per kilo (U$S/Kg)
2010	51,213	4,346,604	84.87
2011	38,845	4,537,727	116.81
2012	41,721	5,401,643	129.47

**Table 3:** Identified compounds in ethanol extracts of propolis.

Sample	Compounds identified	Reference
Bulgarian propolis	3,7-Dihydroxy-5-methoxyflavanone 2,5-dihydroxy-7-methoxyflavanone	[50]

North and South Bulgaria	Dihydrocaffeic acid Dihydroferulic acid Dihydroxyacetophenone hydroxymethoxyacetophenone *β*-Phenethyl alcohol Benzyl alcohol pinobanksin Pinostrobin Dimethyl kaempferol	[51]

Brazil/São Paulo state	3-Prenyl-4-dihydrocinnamoloxynnamic acid	[23]

Brazil/São Paulo state/Botucatu city	9-E and 9-Z 2,2-Dimethyl-6-carboxyethenyl-8-prenyl-2H-benzopyran	[52]

Brazil/São Paulo state	Dehydroabietic acid Abietic acid *β*-Amyrine Triterpenic alcohol of amyrine Lanosterol isomer with 9(11) double bond	[53]

Not reported	(E)-2,3-Dihydroconiferyl p-coumarate (E)-3-{2,3-Dihydro-2-[2-[(E)-pcoumaroyloxy]-1-methylethyl]-5-benzofuranyl}-2-propenoic acid (E)-4-(2,3-Dihydrocinnamoyloxy) cinnamic acid (E)-3-(2,2-Dimethyl-3,4-dihydro-3-hydroxy-2H-1-benzopyran-6-yl)-2-propenoic acid (E)-3-[2,3-Dihydro-2-(1-methylethenyl)-5-benzofuranyl]-2-propenoic acid (E)-3-[2,3-Dihydro-2-(1-methylethenyl)-7-prenyl-5-benzofuranyl]-2-propenoic acid (E)-3-{3-[(E)-4-(2,3-Dihydrocinnamoyloxy)-3-methyl-2-butenyl]-4-hydroxy-5-prenylphenyl}-2-propenoic acid Dihydrokaempferol (aromadendrin) 6-Methoxykaempferol 4-Hydroxy-3-prenylbenzoic acid Plicatin B Capillartemisin A	[54]

Japan/Okinawa	Prokinawan	[55]

Brazilian propolis type 6	Hyperibone A	[56]

Mexico/Champoton	1-(3′,4′-Dihydroxy-2′-methoxyphenyl)-3-(phenyl)propane (z)-1-(2′-Methoxy-4′,5′-dihydroxyphenyl)-2-(3-phenyl)propene 3-Hydroxy-5,6-dimethoxyflavan (−)-7-Hidroxyflavanone (−)-Mucronulatol (−)-Arizonicanol a (+)-Vestitol (−)-Melilotocarpan a (−)-Melilotocarpan d (+)-Pinocembrin	[57]

Greece (six regions)	18-Hydroxyabieta-8, 11,13-triene Dihydroxyabieta-8,11,13-triene; hydroxydehydroabietic acid 18-Succinyloxyabietadiene 18-Succinyloxyabietadiene (isomer) 18-Succinyloxyhydroxyabietatriene	[58]

Kenyan propolis	Tetrahydrojusticidin B 6-Methoxydiphyllin Phyllamyricin C Macarangin Schweinfurthin A Schweinfurthin B	[59]

Indonesia/East Java province/Batu city	5-Pentadecylresorcinol 5-(8′z, 11′z Heptadecadienyl)-resorcional 5-(11′z-Heptadecenyl)-resorcinol 5-Heptadecylresorcional Propolin d Propolin c Propolin f Propolin g	[60]

Jordanian propolis	24(z)-1*β*-3*β*-Dihydroxyeupha-7,24-dien-26-oic acid	[61]

Honduras	(E, Z)-Cinnamyl cinnamate	[62]

Solomon island	Solophenol (A)	[63]

**Table 4:** Propolis of different geographic regions and their principal plants' sources of chemical compounds (from Bankova, 2005).

Geographic origin	Plant source	References
Bulgaria	*Populus nigra, P. italic *	[16, 50, 63]
Albania	*Populus nigra *	[63]
Bulgaria	*Populus tremula *	[16]
Mongolia	*Populus suaveolens *	[16, 63]
USA (mainland)	*Populus fremontii *	[16]
USA (Hawaiian islands)	*Plumeria acuminate, Plumeria acutifolia *	[16]
United Kingdom	*Populus euramericana *	[16]
Hungary	*Betula, Populus, Pinus, Prunus,* and *Acacia *spp*.; Aesculus hypocastane *	[16]
Poland	*Betula, Alnus *spp.	[16]
Equatorial regions	*Delchampia *spp.	[16]
Equatorial regions	*Clusia *spp.	[16, 64]
Australia	*Xanthorrhoea *	[2]
North temperate zone	Poplar, birch, elm, alder, beech, conifer, and horse chestnut	[2]
Europe, North America, nontropic regions of Asia (poplar propolis)	*Populus* spp. of section *Aigeiros*, most often *P. nigra* L.	[1, 34, 65]
Russia (birch propolis)	*Betula verrucosa* Ehrh.	[66]
Brazil (green-alecrim-propolis)	*Baccharis* spp. Predominantly *B. dracunculifolia *DC.	[14, 67]
Cuba, Venezuela	*Clusia minor*	[16, 68–70]
	*Clusia* spp.	
South Brazil (type 3), Argentine, and Uruguay	*Populus alba *	[71]
Brazil (type 6 from northeastern Brazil)	*Hyptis divaricate *	[14]
Brazil (type 13 from northeastern Brazil)	*Dalbergia ecastaphyllum *	[17, 42]

**Table 5:** Classification of Brazilian propolis, according to their physicochemical characteristics and location [17, 41].

Groups	Ethanolic extract of propolis	
	Color	Origin of propolis
Group 1 (RS5)	Yellow	Southern
Group 2 (RS1)	Brown	Southern
Group 3 (PR7)	Dark brown	Southern
Group 4 (PR8)	Brown	Southern
Group 5 (PR9)	Greenish brown	Southern
Group 6 (BA11)	Reddish brown	Northeast
Group 7 (BA51)	Greenish brown	Northeast
Group 8 (PE5)	Dark brown	Northeast
Group 9 (PE3)	Yellow	Northeast
Group 10 (CE3)	Dark yellow	Northeast
Group 11 (PI11)	Yellow	Northeast
Group 12 (SP12)	Green or greenish brown	Southeast
Group 13 (AL)	Red	Northeast

**Table 6:** Chemical constituents of propolis that possess known pharmacological activities.

Chemical compounds	Activities	References
Acacetin	Anti-inflammatory	[72]
Apigenin	Anti-inflammatory	[72]
Artepillin C	Antimicrobial Antitumor activity Antioxidative	[23, 41, 73–75]
Caffeic acid phenethyl ester	Antitumor activity Anti-inflammatory	[76, 77]
Chrysin	Anti-inflammatory	[72]
Caffeic acid	Antibacterial Antifungal Antiviral Anti-inflammatory	[72, 78–80]
Cinnamic acid	Anti-inflammatory	[72]
Dicaffeoylquinic acid derivatives	Hepatoprotective	[81]
Ferulic acid	Anti-inflammatory	[72]
Galangin	Anti-inflammatory	[72]
Gallic acid	Anti-inflammatory	[72]
Moronic acid	Anti-HIV	[9]
Isoferulic acid	Anti-inflammatory	[72]
Pinostrobin	Local anesthesia	[82]
Protocatechuic acid	Anti-inflammatory	[72]
Pinocembrin	Antibacterial Antifungal Antimold Local anesthesia	[79, 80, 82, 83]
Propofol	Antioxidative	[7]
*ρ*-Coumaric acid	Antibacterial	[74]
*m*-Coumaric acid	Anti-inflammatory	[72]
*o*-Coumaric acid	Anti-inflammatory	[72]
Quercetin	Anti-inflammatory Antiviral Antihistamine Ulcer healing Capillary strengthening	[41, 78]
Volatile constituents (phenols, esters, terpenoids, etc.)	Antibacterial	[64]
2,2-Dimethyl-6-carboxyethyl-2H-1-benzopyran	Antimicrobial	[81]
3-[3,4-Dihydroxy-5-prenylphenyl]-2-(E)-propenoic acid	Antioxidative	[84]

**Table 7:** Recent studies on application of propolis in medicine.

Application in medicine			
Geographic origin of propolis	Activity attributed	Test performed	References
Brazil (southern)	Anti-HIV activity	*In vitro* (H9 Lymphocytes)	[9]
Brazil	Anticancer activity	*In vivo*—mice (pulmonary tumors)	[85]
Brazil	Anticancer activity	*In vitro* (human tumor cell lines)	[86]
Brazil (group 3 and group 12)	Suppression of dioxin	*In vitro *	[87]
Chile	Antioxidant and anticancer	*In vitro* (KB cells—human mouth epidermoid carcinoma cells; Caco-2 cells—human colon adenocarcinoma cells)	[88]
Brazil	Anticancer activity	*In vitro* and *in vivo* (retinal damage)	[89]
Brazil (group 3, group 12, and bud resins of botanical origin)	Anticancer activity	*In vitro* (human prostate epithelial cells)	[90]
Brazil	Antiinfluenza virus activity	*In vivo*-mice (influenza virus)	[91]
Jordanian	Antibacterial	*In vitro *	[92]
Tunisia	Anticancer activity	*In vitro* (cancer cell lines—HT29, A549, Hep-2, raw264.7, and Vero)	[93]
Brazil (group 12 and artepellin C)	Immunosuppressant	*In vitro* (CD4 T cell)	[94]
Portugal	Anticancer activity	*In vitro* (human renal cancer)	[95]
Israel (Kibbutz Yad Mordecai and CAPE)	Anticancer activity	*In vitro* (human T-cell lines)	[96]
Brazil	Anticancer activity	*In vitro* (human breast cancer MCF-7 cells)	[97]
Brazil (group 12 and group 13)	Anticancer activity	*In vitro* (human cell lins of leukemia)	[98]
CAPE (derived from honeybee hive propolis)	Anticancer activity	*In vitro* (human prostate cancer cells)	[99]
Brazil	Anticancer activity	*In vivo*—mice (skin carcinogenesis)	[100]
Poland	Anticancer activity	*In vitro* (*U87MG human glioblastoma*)	[101]

**Table 8:** Recent studies on application of propolis in dentistry.

Application in dentistry			
Geographic origin of propolis	Activity attributed	Test performed	References
Japan	Inhibited glucosyltransferase activity	*In vivo*—rats	[102]
Brazil (extracts of propolis from the states of Minas Gerais, São Paulo, Goiás, Mato Grosso do Sul, Paraná, and Rio Grande do Sul)	Inhibited glucosyltransferase activity	*In vitro *	[8]
Brazil (extracts of propolis from the states of Minas Gerais and Rio Grande do Sul)	Cariostatic effect	*In vivo*—rats	[103]
Brazil (extracts of propolis from the states of Minas Gerais and Rio Grande do Sul)	Inhibited glucosyltransferase activity	*In vitro *	[104]
Brazil (Minas Gerais state)	Antibacterial activity Inhibition of cell adherence Inhibition of water-insoluble glucan formation	*In vitro *	[105]
Brazil (extracts of propolis from the states of Minas Gerais and Rio Grande do Sul)	Cariostatic effect	Human	[106]
Brazil (extract of propolis from Bahia state)	Exceptionally effective against *Streptococcus mutans *	*In vitro *	[107]
Apigenin and tt-farnesol	Glucosyltransferase activity	*In vivo*—rats	[108]
Apigenin and tt-farnesol (association)	Cariostatic effect	*In vitro *	[109]
Brazil (Isolated fractions)	Cariostatic effect	*In vitro* and *in vivo *	[110]
Brazil (Bahia state)	Cariostatic effect	*In vivo*—rats	[111]
Brazil (extracts of propolis)	Cariostatic effect	Human	[112]
Tunisia	Cariogenic activity Inhibition oral biofilm formation	*In vitro *	[93]
Korea	Antibacterial activity	*In vitro *	[113]
